# Effect of the “Normalized Epidemic Prevention and Control Requirements” on hospital-acquired and community-acquired infections in China

**DOI:** 10.1186/s12879-021-06886-y

**Published:** 2021-11-23

**Authors:** Caiyun Chen, Ping Zhu, Yongxiang Zhang, Bo Liu

**Affiliations:** 1 Department of Pharmacy, The First Affiliated Hospital of Nanjing Medical University, Nanjing, China; 2 Department of Medical Service, The First Affiliated Hospital of Nanjing Medical University, Nanjing, China; 3 Department of Infection Prevention and Control, The First Affiliated Hospital of Nanjing Medical University, 300 Guangzhou Road, Nanjing, 210029 China; 4 Department of Public Health and Infection Prevention and Control, Ke Zhou People’s Hospital of Nanjing Medical University, Ke Zhou, China

**Keywords:** “Normalized Epidemic Prevention and Control Requirements”, Epidemic prevention and control, Hospital-acquired infection, Infectious sites, Multi-drug resistant organisms, Usage of antibiotics

## Abstract

**Background:**

No studies have yet reported the effect of prevention and control measures, which were implemented to combat COVID-19, on the prevention and control of common HAIs. We aimed to examine the effect of the “Normalized Epidemic Prevention and Control Requirements” (implemented in May 2020) by comparison of hospital-acquired infections (HAIs) and community-acquired infections (CAIs) in China during 2018, 2019, and 2020.

**Methods:**

Data of inpatients before and after implementation of new requirements were retrospectively analyzed, including infection rate, use of alcohol-based hand cleaner, anatomical sites of infections, pathogen species, infection by multi-drug resistant species, and use of different antibiotics.

**Results:**

The HAI rate was significantly higher in 2020 than in 2018 and 2019 (*P* < 0.05), and the CAI rate was significantly higher in 2019 and 2020 than in 2018 (*P* < 0.001). Lower respiratory tract infections were the most common HAI during all years, with no significant changes over time. Lower respiratory tract infections were also the most common CAI, but were significantly more common in 2018 and 2019 than 2020 (*P* < 0.001). There were no changes in upper respiratory tract infections among HAIs or CAIs. Most HAIs and CAIs were from Gram-negative bacteria, and the percentages of fungal infections were greater in 2019 and 2020 than 2018. MRSA infections were more common in 2020 than in 2018 and 2019 (*P* < 0.05). The utilization rate and usage days of antibiotics decreased over time (*P* < 0.001) and the culture rate of microbial specimens before antibiotic usage increased over time (*P* < 0.001).

**Conclusions:**

The new prevention and control requirements provided important benefits during the COVID-19 pandemic. However, their effects on HAIs were not obvious.

## Background

During December 2019, many cases of pneumonia of unknown cause appeared in Wuhan (Hubei Province, China). This disease soon spread to other regions of China and then around the world, becoming a pandemic [1]. The World Health Organization (WHO) declared COVID-19 was a Public Health Emergency of International Concern on 1 February 2020 [2]. Researchers identified the causative pathogen was a species in the *Betacoronavirus* genus that was distinct from other well-known species in this genus, SARS-CoV and MERS-CoV [3, 4]. The World Committee on the Classification of Viruses named this novel virus as severe acute respiratory syndrome coronavirus 2 (SARS-CoV-2) and the WHO named the disease as coronavirus disease 2019 (COVID-19) [5].

It is now established that the virus has a high rate of transmission, and this motivated China to implement stringent prevention and control measures. As of 27 August 2021, there have been more than 2.14-billion confirmed cases of COVID-19 and more than 4.47-million deaths worldwide [6]. Although many countries continue to suffer from this serious pandemic, China has been successful in controlling COVID-19, although it had about 100 thousand cases and more than 4000 deaths in total. With the cessation of the blockade in Wuhan, there was increased focus on prevention and control to prevent imported cases and a rebound of indigenous cases in China. Based on risk assessments, targeted methods of prevention and control were implemented that promoted the resumption of work and production [7].

Hospitals are densely populated places and many patients are immunocompromised, increasing the risk of cross-infection. Hospitals are also necessary for the care of people with severe infections and have a key function in prevention and control during pandemics. Therefore, health administrators in China required hospitals to implement “Normalized Epidemic Prevention and Control Requirements” during May 2020 to improve prevention and control measures [8], such as management of hospitalization, management of the family members of patients, management of visitors, and other policies. These requirements have been in place for more than 1 year. In addition to prevention and control of the COVID-19 pandemic, prevention and control of common hospital-acquired infections (HAIs) was also a focus of these prevention efforts. However, no studies have yet reported the effect of these requirements, which were implemented to combat COVID-19, on the prevention and control of common HAIs. In this study, we examined the relationship of the implementation of the “Normalized Epidemic Prevention and Control Requirements” on HAIs and community-acquired infections (CAIs).

## Materials and methods

The records of hospitalized patients from 1 May to 31 December 31 2020 (study group) and from the same periods of 2018 and 2019 (control group) were retrospectively analyzed. All study subjects were patients at the First Affiliated Hospital with Nanjing Medical University, Nanjing. Data were extracted from the nosocomial infection real time surveillance system. Diagnosis of standard of HAIs was according to the definition of the U.S. Centers for Disease Control and Prevention (CDC) [9]. Ethical approval for this retrospective study was obtained from the local ethics committee of the First Affiliated Hospital with Nanjing Medical University (2021-SR-152).

### “Normalized Epidemic Prevention and Control Requirements”

Numerous requirements for prevention and control of the COVID-19 pandemic were implemented in 1 May 2020. In particular, body temperature and travel records were required for all individuals before entering a hospital, and all individuals were also required to wear masks when entering a hospital. Clinics were required to comply with the principle of one patient and one healthcare worker per room. Each hospitalized patient was accompanied by one dedicated person. Hospitalized patients were educated by healthcare workers regarding methods to be used for disease prevention and control. Hospitalized patients with accompanying persons were generally required to wear masks in the ward, and visitors were not allowed to enter. All healthcare workers were required to wear surgical masks during routine medical procedure. In addition, an expert preventionist trained healthcare workers regarding methods to be used to prevent and control infections two times per month.

### Identification of microorganism and standards of multi-drug resistant organism

Bacterial culture and species identification were performed according to the National Clinical Laboratory Operating Procedures (Fourth Edition) [10]. Pure cultured strains were analyzed using an automated bacterial identification instrument (Vitek COMPACT 2, France, bioMérieuxe), identified using supporting identification cards, and the results were reported according to the requirements of the Clinical Laboratory Standards Institute (CLSI) [11]. Multi-drug resistant organisms (MDROs) were defined according to the 2015 consensus of Chinese experts on the prevention and control of HAIs by multi-drug resistant organisms [12]. These MDROs mainly consisted of methicillin-resistant *Staphylococcus aureus* (MRSA), vancomycin-resistant *Enterococcus* (VRE), carbapenem-resistant *Enterobacteriaceae* (CRE), carbapenem-resistant *Acinetobacter baumannii* (CR-AB), and carbapenem-resistant *Pseudomonas aeruginosa* (CR-PA). The control strains used for quality assurance (*Escherichia coli* ATCC 25922, *P. aeruginosa* ATCC 27853, and *S. aureus* ATCC 25923) were provided by the clinical testing center of the National Health Commission. Colonization and contamination by pathogens were actively prevented, and duplicate strains from the same site of one patient were removed from analysis.

### Research contents

The infection rate, use of quick-drying hand disinfectant, anatomical sites of infection, species of infectious pathogens, HAI rate by MDROs, and use of therapeutic antibiotics were compared for the study and control groups.

### Statistical analysis

A database was established using Excel 2010, and the data were then analyzed using SPSS version 23.0 (SPSS Inc., Chicago, IL). Enumeration data were presented as rate and ratio and analyzed using the χ^2^ test or the exact probability method. Measurement data were presented as means ± SDs if it had a normal distribution and as median with interquartile range (IQR) if it had a non-normal distribution. The two groups were compared using the nonparametric Mann–Whitney U test, and comparisons of distributions were performed using the nonparametric Kruskal–Wallis H test. A *P*-value below 0.05 was considered significant.

## Results

### Infection rates before and after the intervention

There were 62,625 inpatients (48.25% males, 51.75% females) in 2018, 70,091 inpatients (48.50% males, 51.50% females) in 2019, and 59,167 inpatients (48.52% males, 51.48% females) in 2020, and their average ages were 52 ± 19 years (2018), 53 ± 18 years (2019), and 53 ± 19 years (2020). There were no significant differences in these gender ratios or ages. However, the numbers of HAIs during 2020 was greater than during 2019 or 2018 (both *P* < 0.05) and the numbers of CAIs during 2020 was greater than during 2019 or 2018 (both *P* < 0.001) (Table 1).

**Table 1:** Percentages of inpatients with hospital-acquired infections and community-acquired infections among all inpatients from 2018 to 2020

Year	Hospital-acquired infections (%)	Community-acquired infections (%)
2018	1.64% (1024/62,625)	2.66% (1664/62,625)
2019	1.56% (1112/71,190)	3.18% (2266/71,190)
2020	1.82% (1077/59,167)	3.33% (1972/59,167)
χ^2^	13.666	52.791
*P*	**0.001**	** < 0.001**

Numbers in parentheses indicate infected inpatients/total inpatients

Bold values mean *P* values are significant

### Changes in open beds and use of alcohol-based hand cleaner

**Fig. 1:**
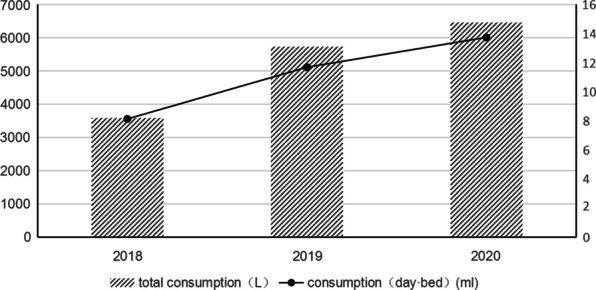
Use of alcohol-based hand cleaner (left axis) and average number of open beds per day (right axis) from 2018 to 2020

The total number of open bed days was 3757 in 2018, 3988 in 2019, and 3820 in 2020, and this corresponded to 8.13 open beds per day in 2018, 11.69 open beds per day in 2019, and 13.75 open beds per day in 2020 (Fig. 1). The total consumption of alcohol-based hand cleaner increased during this time from 3575 mL in 2018, to 5733 mL in 2019, and to 6461 mL in 2020.

### Changes in infection sites

Analysis of the anatomical sites of HAIs indicated the lower respiratory tract was the most common site during all 3 years, with no significant difference among the years (Table 2). However, the percentage of urinary system infections was significantly lower during 2020 than 2018 (*P* = 0.002) and 2019 (*P* = 0.001). The percentage of eye/ear/nose/throat/mouth infections was greater during 2020 than 2018 (*P* = 0.004), but the percentages were similar for 2019 and 2020. Except for infections at “other site” (which were rare during all 3 years), there were no other significant differences.

**Table 2:** Anatomical sites of hospital-acquired infections from 2018 to 2020

Infection site	2018	2019	2020	χ^2^	*P*
Lower respiratory tract	39.22%	39.51%	39.70%	0.059	0.971
Urinary system	14.58%	14.83%	10.51%	12.681	**0.002**
Blood system	14.32%	11.28%	12.91%	5.083	0.079
Surgical site	13.13%	11.83%	14.84%	4.965	0.084
Abdomen and digestive system	7.33%	7.49%	9.62%	5.390	0.068
Upper respiratory tract	5.46%	4.89%	3.85%	3.698	0.165
Skin and soft tissue	3.58%	4.26%	2.81%	3.873	0.144
Eye/ear/nose/throat/mouth	0.94%	1.66%	2.49%	8.660	**0.013**
Genital tract	0.43%	0.08%	0.24%	0.807	0.369
Central nervous system	0	0.79%	0	–	–
Bone and joint	0	0.16%	0.16%	–	–
Cardiovascular system	0	0.08%	0	–	–
Other sites	1.02%	3.15%	2.89%	13.915	**0.001**

Each percentage refers to the percentage of all HAIs during a single year

Bold values mean *P* values are significant

Analysis of the anatomical sites of CAIs also indicated that lower respiratory tract infections were the most common during all 3 years (Table 3). Over time, lower respiratory tract infections, blood stream infections, surgical site infections, and eye/ear/nose/throat infections decreased significantly (all *P* < 0.05). Correspondingly, there were increases in the percentage of urinary system infections, abdominal and digestive system infections, and skin and soft tissue infections (all *P* < 0.05).

**Table 3:** Anatomical sites of community-acquired infections from 2018 to 2020

Infection site	2018	2019	2020	χ^2^	*P*
Lower respiratory tract	49.83%	52.65%	43.06%	44.024	** < 0.001**
Urinary system	18.41%	18.72%	26.94%	59.748	** < 0.001**
Abdomen and digestive system	8.16%	11.83%	11.81%	18.153	** < 0.001**
Skin and soft tissue	6.73%	5.37%	7.27%	7.446	**0.024**
Blood system	6.12%	4.73%	4.44%	6.526	**0.038**
Surgical site	1.98%	1.48%	0.74%	11.586	**0.003**
Eye/ear/nose/throat/mouth	1.93%	0.76%	1.02%	13.013	**0.001**
Upper respiratory tract	1.21%	0.92%	0.83%	1.585	0.453
Central nervous system	0.94%	1.24%	0.60%	5.094	0.078
Bone and joint	0.55%	0.16%	0.37%	4.821	0.090
Genital tract	0.55%	0.44%	0.19%	3.745	0.154
Otitis externa, otitis media	0.50%	0	0	–	–
Cardiovascular system	0.33%	0.36%	0.42%	0.208	0.901
Other sites	2.76%	1.32%	2.31%	11.768	**0.003**

Each percentage refers to the percentage of all CAIs during a single year

Bold values mean *P* values are significant

### Changes in infectious pathogens

Analysis of the pathogens responsible for HAIs indicated that Gram-negative species were the most common during all 3 years, and that *Klebsiella pneumoniae*, *A. baumannii*, and *E. coli* were the top three species (Table 4). Compared with 2018, there were greater percentages of fungal infections during 2019 and 2020.

**Table 4:** Species responsible for hospital-acquired infections from 2018 to 2020

2018		2019		2020	
Species	n (%)	Species	n (%)	Species	n (%)
*Klebsiella pneumoniae*	155 (15.72)	*Klebsiella pneumoniae*	188 (17.84)	*Klebsiella pneumoniae*	222 (17.73)
*Acinetobacter baumannii*	154 (15.62)	*Acinetobacter baumannii*	133 (12.62)	*Escherichia coli*	131 (10.46)
*Escherichia coli*	139 (14.10)	*Escherichia coli*	125 (11.86)	*Acinetobacter baumannii*	130 (10.38)
*Pseudomonas aeruginosa*	87 (8.82)	*Candida albicans*	100 (9.49)	*Candida albicans*	104 (8.31)
*Stenotrophomonas maltophilia*	63 (6.39)	*Pseudomonas aeruginosa*	83 (7.87)	*Pseudomonas aeruginosa*	101 (8.07)
*Enterobacter cloacae*	49 (4.97)	*Enterococcus faecium*	54 (5.12)	*Staphylococcus aureus*	65 (5.19)
*Staphylococcus aureus*	45 (4.56)	*Staphylococcus aureus*	50(4.74)	*Stenotrophomonas maltophilia*	56 (4.47)
*Candida albicans*	38 (3.85)	*Stenotrophomonas maltophilia*	45 (4.27)	*Candida smooth*	54 (4.31)
*Enterococcus faecalis*	35 (3.55)	*Candida smooth*	42 (3.98)	*Enterococcus faecium*	48 (3.83)
*Candida smooth*	27 (2.74)	*Enterobacter cloacae*	41 (3.89)	*Enterobacter cloacae*	45 (3.59)
Others	194 (19.68)	Others	193 (18.31)	Others	296 (23.64)
Total	986 (100)	Total	1054 (100)	Total	1252 (100)

Gram-negative bacteria were also responsible for most CAIs during all 3 years, and *E. coli, K. pneumoniae, A. baumannii*, and *P. aeruginosa* were the top four species (Table 5). Compared with 2018, there were also higher percentages of fungal infections during 2019 and 2020, and a decline in the percentage of *S. aureus* infections from 8.96% (2018), to 5.98% (2019), and then to 4.32% (2020).

**Table 5:** Species responsible for community-acquired infections from 2018 to 2020

2018		2019		2020	
Species	n (%)	Species	n (%)	Species	n (%)
*Escherichia coli*	247 (17.85)	*Escherichia coli*	366 (19.38)	*Escherichia coli*	356 (16.71)
*Klebsiella pneumoniae*	190 (13.73)	*Klebsiella pneumoniae*	245 (12.97)	*Klebsiella pneumoniae*	323 (15.16)
*Acinetobacter baumannii*	183 (13.22)	*Acinetobacter baumannii*	178 (9.42)	*Acinetobacter baumannii*	215 (10.09)
*Pseudomonas aeruginosa*	135 (9.75)	*Pseudomonas aeruginosa*	154 (8.15)	*Pseudomonas aeruginosa*	162 (7.61)
*Staphylococcus aureus*	124 (8.96)	*candida albicans*	149 (7.89)	*Candida albicans*	161 (7.56)
*Candida albicans*	65 (4.70)	*Staphylococcus aureus*	113 (5.98)	*Staphylococcus aureus*	92 (4.32)
*Stenotrophomonas maltophilia*	50 (3.61)	*Enterococcus faecium*	71 (3.76)	*Enterococcus faecium*	85 (3.99)
*Enterococcus faecium*	48 (3.47)	*Enterococcus faecalis*	63 (3.34)	*Enterobacter cloacae*	72 (3.38)
*Enterobacter cloacae*	48 (3.47)	*Candida smooth*	53 (2.81)	*Stenotrophomonas maltophilia*	64 (3.00)
*Enterococcus faecalis*	28 (2.02)	*Enterobacter cloacae*	51 (2.70)	*Enterococcus faecalis*	60 (2.82)
others	266 (19.22)	*Stenotrophomonas maltophilia*	46 (2.44)	*Candida smooth*	59 (2.77)
Total	1384 (100)	Total	1889 (100)	Total	2130 (100)

### Changes in HAIs by MDROs

Analysis of HAIs by MDROs indicated MRSA infections were more common in 2020 than in 2018 and 2019 (both *P* < 0.05), but there were no significant changes in infections by VRE, CRE, CR-AB, or CR-PA (Table 6).

**Table 6:** Hospital-acquired infections by the five main MDROs from 2018 to 2020

Year	MRSA		VRE		CRE		CR-AB		CR-PA	
	N	%	n	%	n	%	n	%	n	%
2018	25	0.04%	0	0	5	0.01%	99	0.16%	29	0.05%
2019	25	0.04%	1	0.001%	5	0.01%	87	0.12%	28	0.04%
2020	41	0.07%	1	0.001%	5	0.01%	74	0.13%	39	0.07%
χ^2^	9.038		–		–		3.779		4.813	
*P* value	**0.011**		–		–		0.151		0.09	

Each percentage refers to the percentage of all HAIs during a single year

Bold values mean *P* values are significant

MDRO, multi-drug resistant organism

### Changes in use of different antibiotics

We also determined the rate of antibiotic use as the percentage of all inpatients who received an antibiotic during each year (Table 7). The results indicated a significant decline over time (2018 vs. 2019: *P* = 0.001; 2018 vs. 2020: *P* < 0.001; 2019 vs. 2020: *P* < 0.001). There was also a significant increase in the culture rate of microbial specimens before administration of antibiotics (2018 vs. 2019: *P* < 0.001; 2018 vs. 2020: *P* < 0.001; 2019 vs. 2020: *P* < 0.001) and a significant decrease in the total days of antibiotic use based on Z values (2018 vs*.* 2019: − 16.562; 2018 vs. 2020: − 22.682; 2019 vs. 2020: − 6.670, *P* < 0.001).

**Table 7:** Antibiotic administration practices from 2018 to 2020

Year	Overall (%)	Culture before use (%)	Average days (IQR)
2018	27.08	51.71	6 (3, 9)
2019	26.26	59.28	5 (2, 8)
2020	22.83	62.70	4 (2, 7)
χ^2^	326.538	406.463	600.335
*P*	** < 0.001**	** < 0.001**	** < 0.001**

‘Overall’ is the percentage of all inpatients who received an antibiotic during a single year; ‘Culture before use’ is the percentage of antibiotic users who had cultures taken before use; ‘Average days’ is the average duration of use among antibiotic users. IQR, interquartile range

Bold values mean *P* values are significant

## Discussion

Infection prevention and control has played key roles in the fight against the COVID-19 pandemic, as indicated by the absence of any infections among the nearly 42,000 medical support staff in Hubei Province. The simultaneous increase in the public's awareness of infection prevention and control measures was also successful in preventing COVID-19 in the community [13]. Although China's domestic policies were successful, the pandemic is still a serious problem in many other countries, and China still has a risk that new cases will be imported. The “Normalized Epidemic Prevention and Control Requirements” measures of May 2020 have been key to the control of the COVID-19 pandemic [7, 8]. Based on risk classification, targeted measures should be taken, such as wearing masks in densely closed places. To prevent routine infection and improve infection control, hospitals have taken additional epidemic prevention and control measures, especially wearing masks and strengthening the regulations regarding individuals entering and exiting hospitals.

It is now more than 1 year after implementation of the “Normalized Epidemic Prevention and Control Requirements”, and this study showed that the HAI rate in 2020 (1.82%) was similar to the average HAI rate reported during the most recent 5 years in China (1.94%) [14], but significantly higher than the HAI rates at our facility during 2018 and 2019. Although the CAI rates in 2020 and 2019 were significantly higher than in 2018, there was no difference between 2019 and 2020. Our results indicated the lower respiratory tract was the most common site of HAIs, were similar to the results previously reported for Beijing [15] and in a 5-year national cross-sectional survey [16]. This may be because the respiratory tract is more vulnerable to HAIs, and because it is easy to access the respiratory tract for collection of specimens and diagnosis. We found no significant differences in the percentage of lower respiratory tract HAIs from 2018 to 2020.

From the perspective of epidemic prevention and control, measures such as wearing masks and maintaining a proper social distance are important for prevention of respiratory infectious diseases. However, the rates of HAIs of the respiratory system did not change during our 3-year study period. In addition, measures such as fixed escort, restricted visits, and public education [17, 18] can reduce the risk of cross-infection, but our results provided no evidence that they prevented HAIs. There is a general consensus that increased hand hygiene compliance can reduce the incidence of HAIs [19, 20] and that overall use of alcohol-based hand cleaner is a reliable indicator of hand hygiene compliance [21, 22]. We found that the use of hand cleaner and average number of open beds per day increased in tandem from 2018 to 2020, but this apparently had no impact on the rate of HAIs.

Analysis of the causes and occurrence of HAIs can be difficult [23] because of the impact of endogenous and exogenous factors. Thus, even if exogenous factors are controlled through prevention and control measures, the effects of endogenous factors may remain. Moreover, as a regional comprehensive medical center, our institution mostly admits emergency and critical patients, and a patient's condition determines the risk of a HAI, but the prevention and control measures at the hospital do not.

Our analysis of the anatomical sites of CAIs indicated the lower respiratory tract was the most common site (as with HAIs), but the percentage of lower respiratory tract infections was lower in 2020 than during the previous 2 years. This may be related to the implementation of the “Normalized Epidemic Prevention and Control Requirements” at the community level. In particular, the COVID-19 pandemic forced people to avoid unnecessary socialization, to wear masks, and to perform frequent hand washing to reduce the probability of cross-infection. However, our analysis of upper respiratory tract CAIs indicated no significant change over time. This may be because most upper respiratory tract infections are caused by viruses, and these patients often recover without the need for hospitalization. Therefore, our percentage of CAIs of the upper respiratory tract among inpatients (less than 2%) was probably much lower than the percentage in all community-dwelling individuals.

The composition of pathogens responsible for HAIs was relatively stable from 2018 to 2020. Gram-negative bacteria accounted for nearly 60% of the top ten species, and *K. pneumoniae* ranked first; in contrast, other studies in China reported that *P. aeruginosa* ranked first among HAIs [14, 16, 24]. This difference may be due to geographic differences. The pathogens responsible for CAIs were similar to those responsible for HAIs (i.e., mainly Gram-negative bacteria), but *E. coli* ranked first for CAIs. This is probably because the urinary system was a much more common infection site for CAIs. It is particularly noteworthy that proportion of fungal CAIs and HAIs increased from 2018 to 2020. This may be due to the increase of opportunistic infections caused by the increasing incidence of tumor diseases and use of immunosuppressants [25]. Our analysis of MDROs indicated no significant changes, except that MRSA infection was significantly greater during 2020 than 2018 and 2019. This may be because HAIs by MDROs mostly occur in the ICU [26], and most of these patients have acute and critical diseases, poor clinical status, and the measures of the “Normalized Epidemic Prevention and Control Requirements” had no impact in preventing infections by MDROs in this specific population.

The rational use of antibiotics is closely related to patient prognosis and the occurrence by antibiotic-resistant bacteria. The problem of drug-resistant bacteria has become a serious worldwide public health problem. The frequency of antibiotic use, the culture rate of microbial specimens, and duration of antibiotic use are all important indicators [27]. Our results showed that from 2018 to 2020, the rate and duration of use gradually decreased, and the rate of culturing before use had a gradual increase. Although the utilization rate decreased, administration of antibiotics to more than 20% of all inpatients was still very high because the sum of all HAIs and CAIs among inpatients was less than 5%. At the same time, the significant declines in the utilization rate and days of utilization may be related to changes in Chinese medical insurance policies, in addition to the increased awareness by doctors of the problem of over-prescribing antibiotics.

Of course, there are some limitations in this study. First, the study period was only 3 years, and may not be fully representative of other time periods. Second, this study was conducted at a single center study, and the research area and scope need to be expanded to verify the generalizability of the conclusions. Third, there are many factors that affect specific HAIs, such as MRSA, in-hospital flu, and in-hospital Clostridioides infections. We examined the impact of an intervention on HAIs overall, without specifically examining different types of infections. A more detailed analysis of different types of infections should be further explored in the future.

## Conclusions

We compared HAIs and CAIs in a study group (May to December 2020) with a control group (May to December of 2018 and 2019), and examined all inpatients from the same institution to reduce bias. We found that adoption of the “Normalized Epidemic Prevention and Control Requirements” on 1 May 2020 had no obvious impact on the overall rate of HAIs nor on different parameters related to HAIs.

## Data Availability

After publication, the data generated in the current study will be available to others on reasonable request to the corresponding author.

## Abbreviations

WHO: World Health Organization
COVID-19: Coronavirus disease 2019
SARS-CoV-2: Severe acute respiratory syndrome coronavirus 2
HAIs: Hospital-acquired infections
CAIs: Community-acquired infections
CDC: Centers for Disease Control and Prevention
CLSI: Clinical Laboratory Standards Institute
MDROs: Multi-drug resistant organisms
MRSA: Methicillin-resistant *Staphylococcus aureus*
VRE: Vancomycin-resistant *Enterococcus*
CRE: Carbapenem-resistant *Enterobacteriaceae*
CR-AB: Carbapenem-resistant *Acinetobacter baumannii*
CR-PA: Carbapenem-resistant *Pseudomonas aeruginosa*
IQR: Interquartile range

## References

[1] Contini C, Di Nuzzo M, Barp N (2020). The novel zoonotic COVID-19 pandemic: an expected global health concern. J Infect Dev Countries.

[2] World Health Organization. Coronavirus disease (COVID-19) outbreak (http://www.who.int).

[3] Ludwig S, Zarbock A (2020). Coronaviruses and SARS-CoV-2: a brief overview. Anesth Analg.

[4] Wang Y, Grunewald M, Perlman S (2020). Coronaviruses: an updated overview of their replication and pathogenesis. Methods Mol Biol.

[5] Guo YR, Cao QD, Hong ZS (2020). The origin, transmission and clinical therapies on coronavirus disease 2019 (COVID-19) outbreak-an update on the status. Mil Med Res.

[6] https://covid19.who.int/. Accessed 27 Aug 2021.

[7] Further notice on COVID-19 prevention and control work in key units, key populations and key places. Joint prevention and control mechanism of the State Council; 2020.http://www.gov.cn/zhengce/content/2020-04/08/content_5500241.htm. Accessed 27 Aug 2021.

[8] Notice on implementing the requirements for normalized epidemic prevention and control requirements and further strengthening the infection prevention and control work in medical institutions. Joint prevention and control mechanism of the State Council; 2020. http://www.gov.cn/xinwen/2020-05/01/content_5508135.htm. Accessed 27 Aug 2021.

[9] Horan TC, Andrus M, Dudeck MA (2008). CDC/NHSN surveillance definition of health care-associated infection and criteria for specific types of infections in the acute care setting. Am J Infect Control.

[10] Hong S, Yusan W, Ziyu S (2015). National clinical laboratory operating procedures.

[11] Clinical Laboratory Standard Institute (CLSI). Performance standards for antimicrobial susceptibility testing (2017) CLSI approved standards CLSI M100-S23. Wayne, PA: CLSI; 2017.

[12] Huang X, Deng Z, Ni Y, et al. Chinese experts, consensus on prevention and control of multidrug resistance organism healthcare-associated infection. Chin J Infect Control. 2015;14(1): 1–9. 2008.03.002.

[13] Zhao Y, Cheng S, Yu X (2020). Chinese public's attention to the COVID-19 epidemic on social media: observational descriptive study. J Med Internet Res.

[14] Ling-fang LV, Zhang HY, Zhou CL (2020). Analysis of surveillance data of nosocomial infections in a level A tertiary hospital from 2014 to 2018. Chin J Disinfect.

[15] Yao H, Huo R, Liu Y (2019). Incidence of healthcare-associated infections in a tertiary hospital in Beijing, China: results from a real-time surveillance system. Antimicrob Resist Infect Control.

[16] Ren N, Wen X, Wu A (2016). Nationwide cross-sectional survey on healthcare-associated infection in 2014. Chin J Infect Control.

[17] Cawcutt KA, Marcelin JR, Silver JK (2019). Using social media to disseminate research in infection prevention, hospital epidemiology, and antimicrobial stewardship. Infect Control Hosp Epidemiol.

[18] Siracusa M, Scuri S, Grappasonni I (2019). Healthcare acquired infections: malpractice and litigation issues. Ann Ig.

[19] Fredj SB, Cheikh AB, Bhiri S (2020). Multimodal intervention program to improve hand hygiene compliance: effectiveness and challenges. J Egypt Public Health Assoc.

[20] Gutierrez J, Alloubani A, Alzaatreh M (2021). Impact of an interventional program on improving compliance of hand hygiene and reducing hospital-acquired infection in the critical care unit. J Glob Infect Dis.

[21] Wetzker W, Bunte-Schönberger K, Walter J (2017). Use of ventilator utilization ratio for stratifying alcohol-based hand-rub consumption data to improve surveillance on intensive care units. J Hosp Infect.

[22] Kramer TS, Walter J, Schröder C (2021). Increase in consumption of alcohol-based hand rub in German acute care hospitals over a 12 year period. BMC Infect Dis.

[23] Dalton KR, Rock C, Carroll KC (2020). One Health in hospitals: how understanding the dynamics of people, animals, and the hospital built-environment can be used to better inform interventions for antimicrobial-resistant gram-positive infections. Antimicrob Resist Infect Control.

[24] Sheng B, Ye Y, Li J (2017). Nosocomial infection: an analysis and investigation of 70160 inpatients cases. Anhui Med Pharm J.

[25] Pathakumari B, Liang G, Liu W (2020). Immune defence to invasive fungal infections: a comprehensive review. Biomed Pharmacother.

[26] De Waele JJ, Boelens J, Leroux-Roels I (2020). Multidrug-resistant bacteria in ICU: fact or myth. Curr Opin Anaesthesiol.

[27] Karanika S, Paudel S, Grigoras C (2016). Systematic review and meta-analysis of clinical and economic outcomes from the implementation of hospital-based antimicrobial stewardship programs. Antimicrob Agents Chemother.

